# Synchronous “skip” facial metastases from colorectal adenocarcinoma: a case report and review of literature

**DOI:** 10.1186/s12876-022-02141-z

**Published:** 2022-02-16

**Authors:** A. Picciariello, G. Tomasicchio, G. Lantone, G. Martines, R. Dibra, G. Trigiante, A. d’Amati, D. Piscitelli, D. F. Altomare

**Affiliations:** 1 Deparment of Emergency and Organ Transplantation, University “Aldo Moro” of Bari, Piazza G Cesare, 11, 70124 Bari, Italy; 2 Section of Pathology, Department of Emergency and Organ Transplantation (DETO), University of Bari “Aldo Moro”, Bari, Italy; 3 IRCCS Istituto Tumori Giovanni Paolo II, Bari, Italy

**Keywords:** Skin metastasis, Colorectal cancer, Facial lesions, Colorectal surgery

## Abstract

**Background:**

Skin metastases from colorectal adenocarcinoma are rare conditions that are metachronous in most of cases and may represent the first sign of a recurrence. These lesions are usually located to the abdominal wall on postoperative scars, perineum and chest due to direct spread from the tumor or to the lymphatic and venous dissemination. We describe a rare case of synchronous skin metastases in a patient affected by sigmoid adenocarcinoma with no sign of liver and lung repetitive lesions.

**Case presentation:**

We admitted a 59 years old male, with no relevant medical history. He was evaluated by our tertiary center of colorectal surgery complaining diarrhoea and abdominal pain. The physical examination revealed a palpable mass in left flank of the abdomen. The colonoscopy showed a sub-stenosis of the sigmoid colon (G2 adenocarcinoma). No repetitive lesions were detected by the preoperative CT scan. The patient reported a rapid grow of a soft supralabial and chin nodules in the last 2 months, which he believed to be related to the use of the mask due to COVID-19 pandemic. A laparoscopic left hemicolectomy with complete mesocolic excision and a local excision of both facial nodules were performed. The histological examination revealed a poorly differentiated signet ring cell colorectal adenocarcinoma with metastases in seven pericolic lymphonodes. The excisional biopsy of the skin nodules revealed a subcutaneous metastases from primary colorectal tumour.

**Conclusions:**

As far as we know, synchronous facial metastases from colorectal cancer in the absence of any other metastases has never been described before. The onset of new skin nodules in patients affected by colorectal cancer should raise-up the clinical suspicion of metastatic lesions even when repetitive lesions are not detected in the liver or lungs.

## Background

Skin metastases from colorectal adenocarcinoma are rare conditions occurring in less than 5% of patients with a poor prognosis [1, 2].

Metastases of the skin are usually metachronous with a clinical manifestation after an average period of 4.9 years following the excision of the primary tumour [3] and they involve the abdominal wall on the postoperative scars, perineum and chest [4, 5]. These lesions could be the first sign of a recurrence and their location could be due to direct spread from the neoplasm or lymphatic dissemination or, in case of distant metastases to the venous invasion [6].

Here we present a rare case of synchronous facial metastases in a patient with sigmoid adenocarcinoma with no repetitive lesions in liver and lungs.

## Case presentation

A 59 years old patient was evaluated in our tertiary centre of colorectal surgery complaining diarrhoea and a palpable mass on the left quadrant of the abdomen. The colonoscopy demonstrated a sub-stenosis of the sigmoid colon due to a cancer which was histologically examined (G2 adenocarcinoma of the colon). Patient medical history was silent with no intake of medications and no previous surgery.

**Fig. 1:**
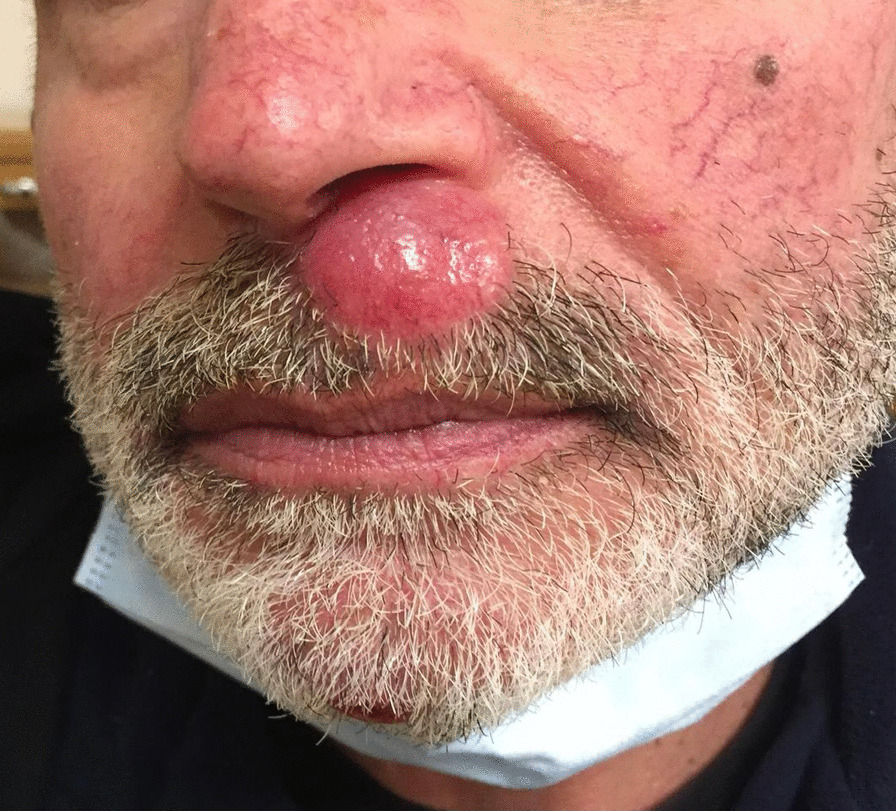
Clinical aspect of supralabial and chick nodules

During the general physical examination of the patient a soft nodule of 2 cm of diameter was observed on the supralabial region and another nodule of 0.5 cm was found on the chin (Fig. 1). The patient reported a rapid grow of the supralabial nodule in the last 2 months which he believed to be correlated with use of the mask due to COVID-19 pandemic. At the ultrasound both nodules appeared as a hypoechoic mass with peripheral pseudo capsule. The staging of the primitive sigmoid carcinoma by abdominal and thoracic CT scan with contrast medium did not detect any metastatic lesion in the liver and lungs.

Serum concentrations of carcinoembryonic antigen (CEA) and alpha-fetoprotein (AFP) levels were within normal ranges.

A laparoscopic left hemicolectomy with complete mesocolic excision and a local excision of both facial nodules were performed during the same operation by two surgical teams. The postoperative course was uneventful and the patient was discharged five days after surgery without complication.

The histological examination of the resected intestinal segment revealed a poorly differentiated signet ring cell colorectal adenocarcinoma extending through the full thickness of the colonic wall and invading the pericolic soft tissues and the visceral layer of the peritoneum.

**Fig. 2:**
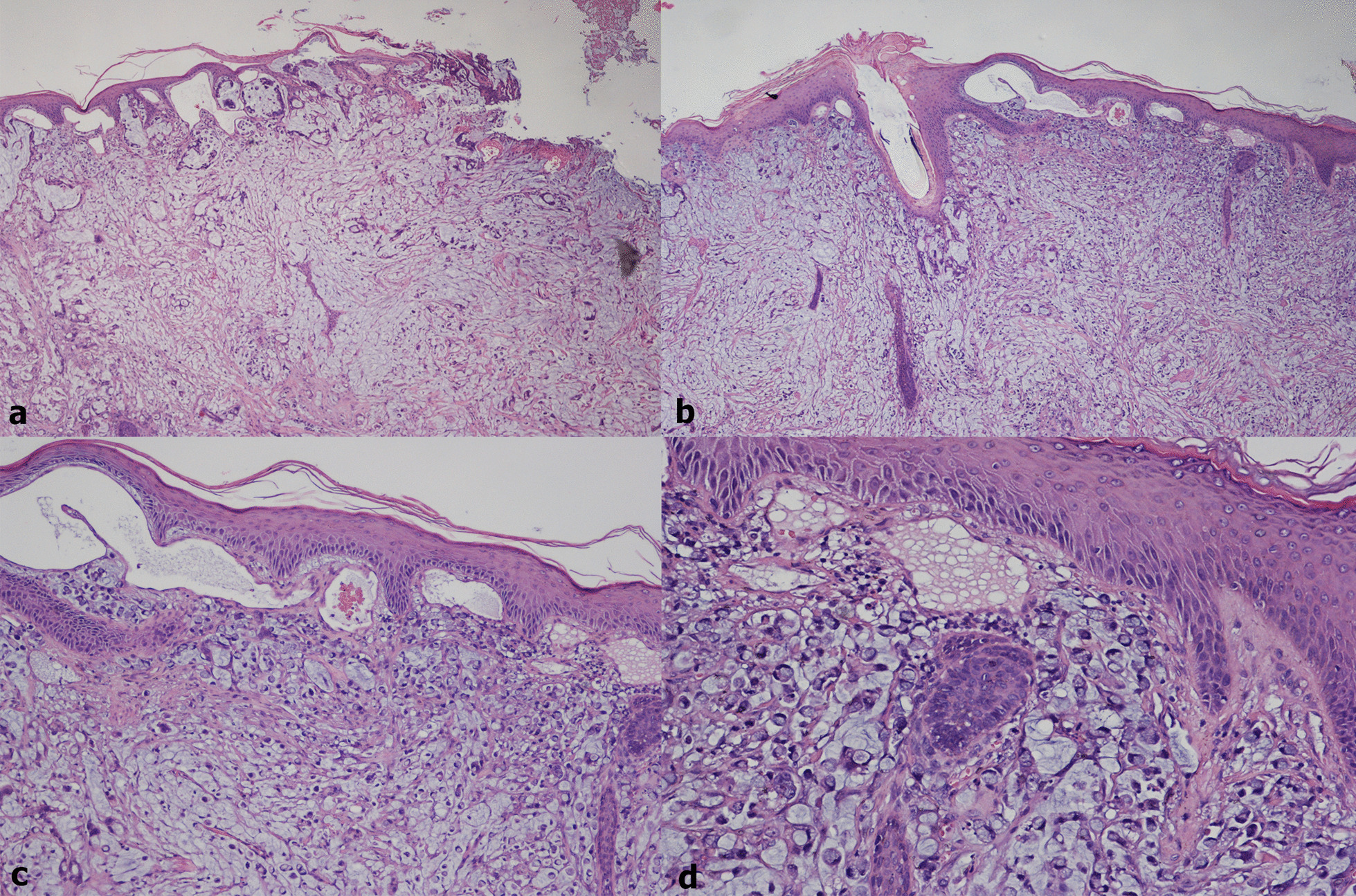
Cutaneous metastases of signet-ring cell colorectal adenocarcinoma (hematoxylin–eosin; **a**, **b** 40x; **c** 100x; **d** 200x)

Seven out of twenty lymph nodes, isolated from the pericolic soft tissues, showed metastases from signet ring cell adenocarcinoma. The excisional biopsy of the skin nodules revealed a dermal and subcutaneous infiltration of malignant signet ring cells floating in mucin lakes, along with focal epidermal ulceration, confirming the metastatic nature of this cutaneous lesions (Fig. 2).

**Fig. 3:**
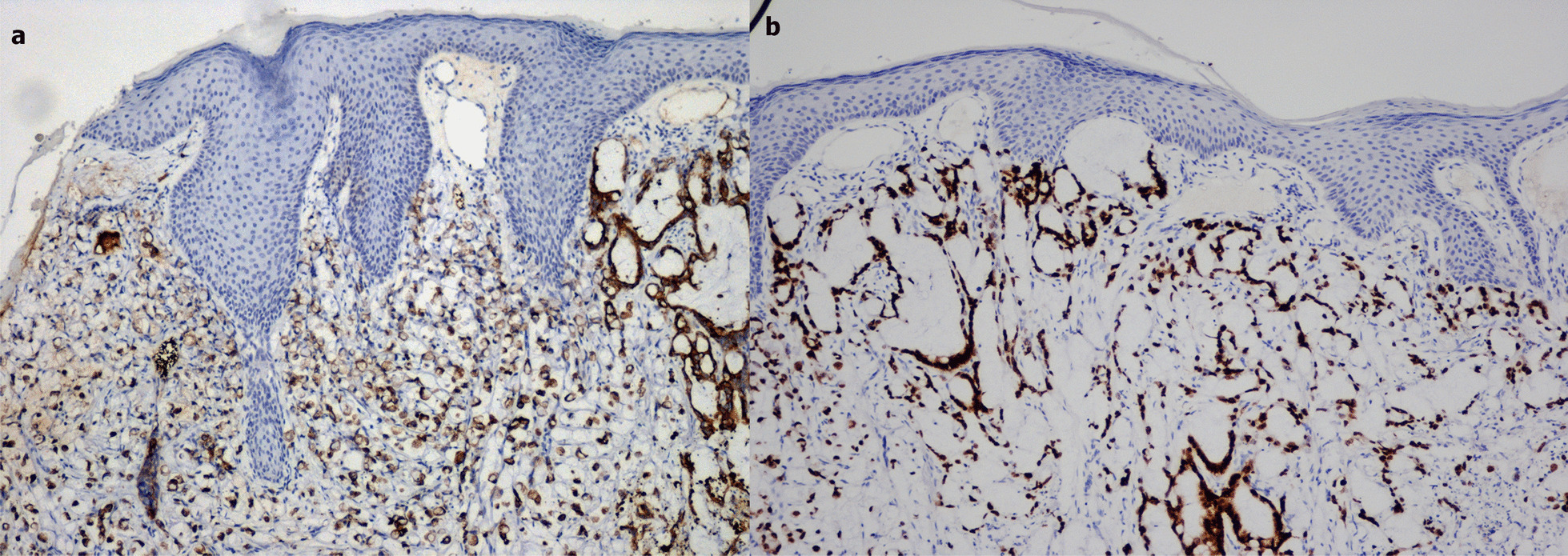
Immunohistochemical results. **a** neoplastic cells positivity for CK20 (100x). **b** neoplastic cells positivity for CDX2 (100x)

Both in the primary and in the metastatic sites, the neoplastic cells expressed positivity for CK20 and CDX2 on immunohistochemistry (Fig. 3).

**Fig. 4:**
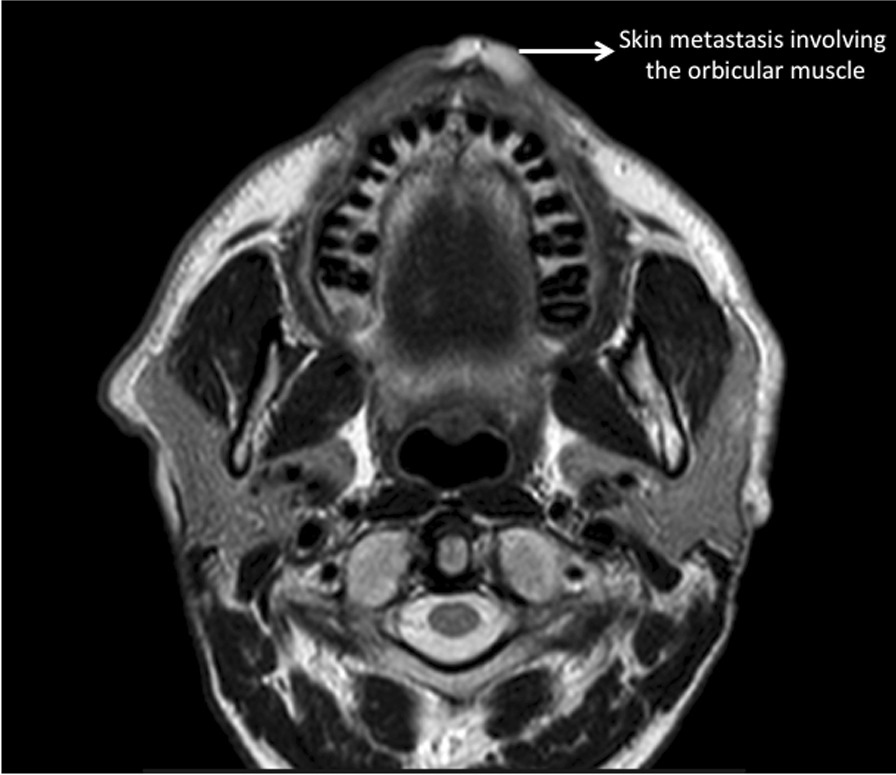
Facial MRI showing an involvement of the orbicular muscle by the metastatic process

Postoperative facial MRI showed an involvement of the orbicular muscle by the metastatic process (Fig. 4). No metastases were detected at the MRI of the liver performed one month after surgery.

After an interdisciplinary team consultation, the patient started the first cycle of adjuvant chemotherapy with FOLFOXIRI regimen.

**Fig. 5:**
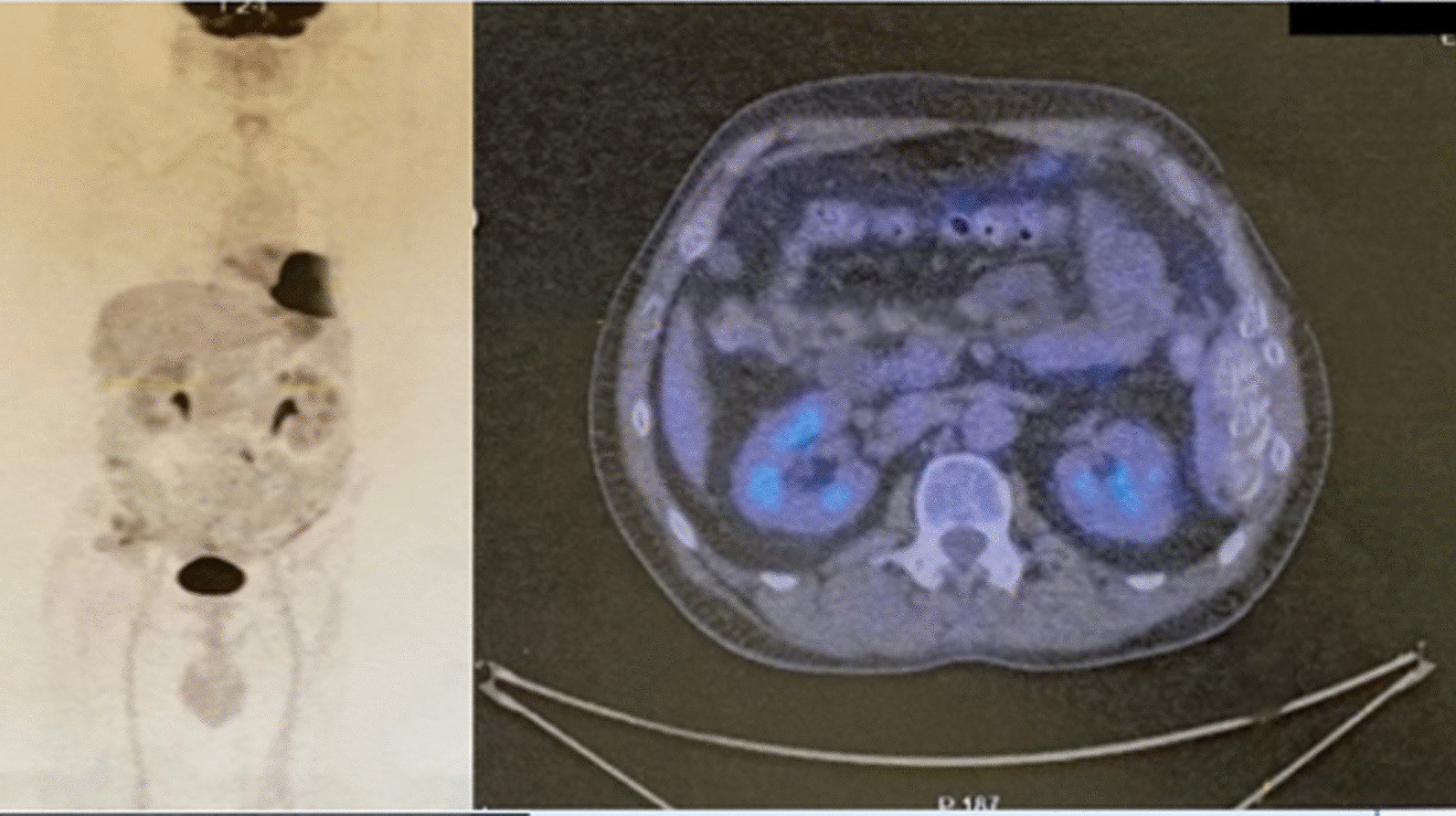
PET performed 2 months after start of the chemotherapy, with no detection of further metastatic lesions

As requested by the oncologist, a total body positron emission tomography with 2-deoxy-2-[fluorine-18]fluoro-D-glucose integrated with computed tomography (18F-FDG PET/CT).

was performed 2 months after the start of chemotherapy with no detection of further metastatic lesions (Fig. 5).

**Fig. 6:**
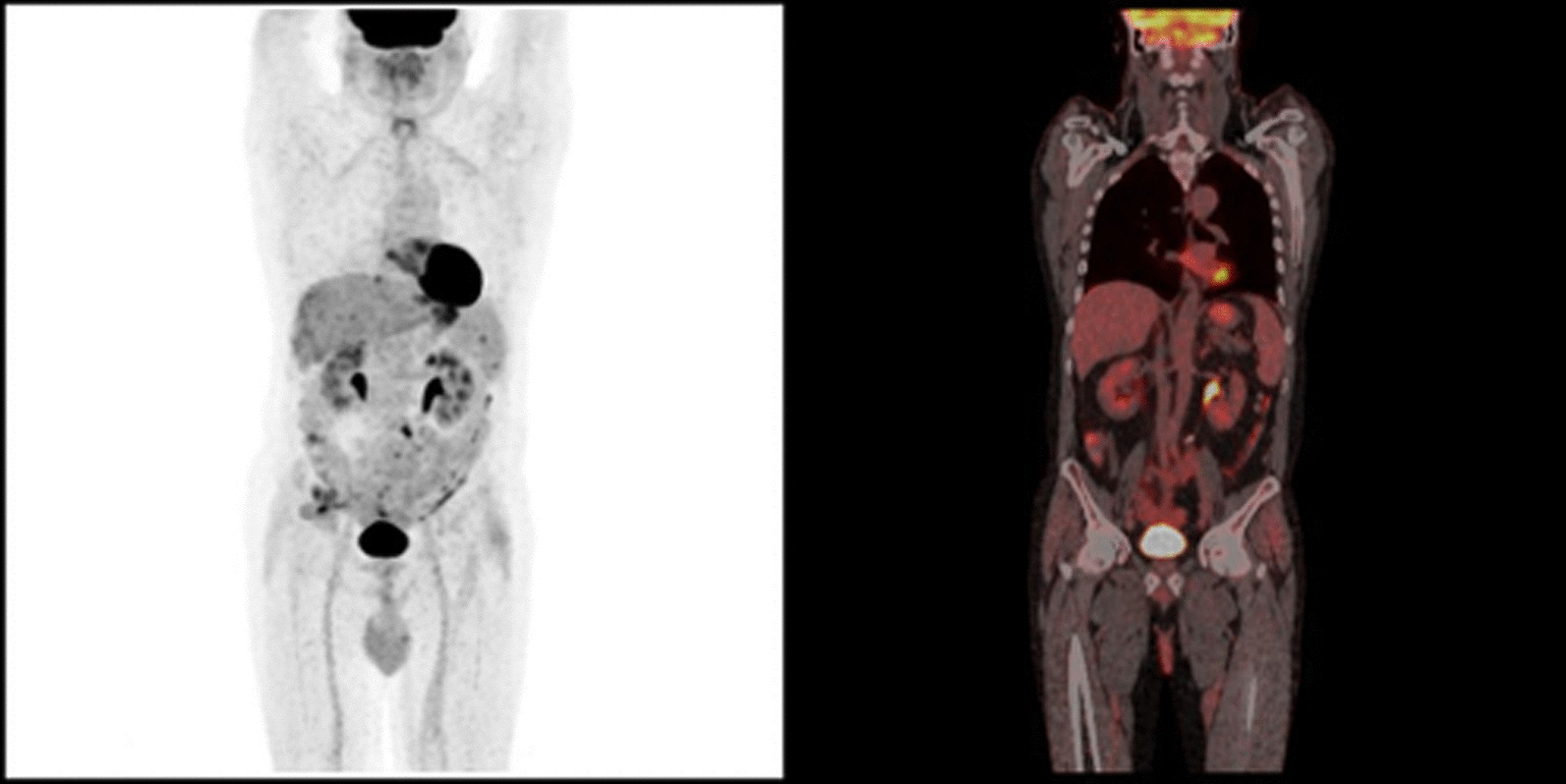
PET performed 6 months after surgery showing the onset of a new metastatic process

Due to the worsening of the clinical condition a further 18F-FDG PET/CT was performed after six months showing a pathological signal (maximum standardized uptake value: 5.5) in left mesogastric paramedian region, in the left flank and in the presacral region with an involvement of the peritoneum (Fig. 6).

## Discussion and conclusion

Facial skin metastasis from colorectal adenocarcinoma are extremely uncommon and occur in 0.5% of patients already affected by metastatic cancer [7].

The usual clinical aspect of a facial metastasis is a nodule with signs of ulceration or a fibrotic painless process [8]. As reported in literature, in all of the cases, patients affected by skin metastases from colorectal adenocarcinoma already have liver or lung repetitive lesions at the time of the diagnosis [9] or skin metastases arise after cancer removal as a metachronous cancer recurrence [10].

Immunohistochemistry can play a pivotal role in the characterization of skin metastases from colon cancer. In fact the expression of the cytokeratin (CK) 20 and the absence of CK 7 is really helpful for the diagnosis of colorectal adenocarcinoma [11, 12].

Life expectancy in patients with cutaneous and subcutaneous metastases from primary colorectal tumour is short with a median survival ranging from 4.4 to 18 months as reported in literature [7, 13].

Compared to the other cases reported in literature, our case differs due to the unusual presentation of the nodules and the absence of metastases in liver and lungs which are most commonly involved before other sites.

As far as we know, synchronous facial metastases from colorectal cancer in the absence of any other metastases has never been described before. Nevertheless the onset of new skin nodules in patients affected by colorectal cancer should raise-up the clinical suspicion of metastatic lesions even when repetitive lesions are not detected in the liver or lungs. The use of 18F-FDG PET/CT could lead to improve the staging and restaging of disease with a direct impact on patient management and survival.

## Data Availability

If requested data and material used for this case report will be provided.
